# Joint Masked Face Recognition and Temperature Measurement System Using Convolutional Neural Networks

**DOI:** 10.3390/s23062901

**Published:** 2023-03-07

**Authors:** Tsung-Han Tsai, Ji-Xiu Lu, Xuan-Yu Chou, Chieng-Yang Wang

**Affiliations:** Department of Electrical Engineering, National Central University, No.300, Jung-Da Rd., Taoyuan City 320317, Taiwan

**Keywords:** face detection, masked face recognition, deep learning, embedded system

## Abstract

With the outbreak of COVID-19, epidemic prevention has become a way to prevent the spread of epidemics. Many public places, such as hospitals, schools, and office places, require disinfection and temperature measurement. To implement epidemic prevention systems and reduce the risk of infection, it is a recent trend to measure body temperature through non-contact sensing systems with thermal imaging cameras. Compared to fingerprints and irises, face recognition is accurate and does not require close contact, which significantly reduces the risk of infection. However, masks block most facial features, resulting in the low accuracy of face recognition systems. This work combines masked face recognition with a thermal imaging camera for use as an automated attendance system. It can record body temperature and recognize the person at the same time. Through the designed UI system, we can search the attendance information of each person. We not only provide the design method based on convolutional neural networks (CNNs), but also provide the complete embedded system as a real demonstration and achieve a 94.1% accuracy rate of masked face recognition in the real world. With the face recognition system combined with a thermal imaging camera, the purpose of screening body temperature when checking in at work can be achieved.

## 1. Introduction

In recent years, the attendance system, which refers to a management system to record the attendance of employees, has grown into a friendlier operation. There are many types of attendance systems, such as time clocks, inductive clocking, and biometric authentication systems. Among them, biometric authentication records attendance through fingerprints [1], face recognition [2,3], etc. Biometric identification through the unique characteristics of each person is operated without the need to carry any form of key. This method can not only solve the doubt that the time card will be used by others, but also avoid the problem of the loss of the card. Compared with other methods of biological characteristics, face recognition is the best because it does not require contact or additional actions. Unlike fingerprint or palm recognition, face recognition does not require the trouble of reaching out for recognition, and it also reduces the hygiene problem of multiple people touching the sensor.

The rapid development of artificial intelligence (AI) and machine learning has driven many products to apply biometric recognition to attendance systems, such as fingerprint recognition, palm recognition, voiceprint recognition, and retinal recognition. The difference in biological characteristics between individuals is used as the basis for discrimination to achieve the effect of access control.

In the AI era, CNNs have been successfully applied to computer vision, such as image classification [4], face recognition [5,6], and image segmentation [7,8]. The success of AI is mainly attributed to the advancement of deep architecture, GPU computing, and large training datasets. As a result, it has led to achievements in face recognition. At present, computers can already perform better than humans in these aspects [9,10].

However, face recognition is quite complicated, and the face of a person is quite variable. Its performance depends on angle, position, light source, hairstyle, expression, lens factor, etc., which will cause considerable differences in the input image.

Recently, due to the spread of COVID-19 around the world, masks have become indispensable items in our lives. Governments encourage people to wear masks to cover their mouths and nose in public to prevent the spread of infection. However, the use of masks poses a huge challenge to face recognition systems [11], because most of the facial features are blocked. Alzu’bi et al. [12] showed that MFR has become a hot area with many relevant retrospective studies on masked face recognition (MFR). To achieve a non-contact attendance system, masked face recognition is needed. The design based on CNN always achieves a high recognition rate based on various datasets. However, the existing dataset cannot be applied to faces wearing masks, so a more intuitive approach is necessary to add masks to existing datasets and train a neural network that can maintain high accuracy even when the mouth and nose are covered by masks.

In addition, a set-up action for people to carry out temperature measurements is very time-consuming and can put people at risk of infection. At present, there are many techniques to apply thermal imaging cameras to perform rapid screening of body temperature. These devices are mostly set up and installed at the entrances or exits of public areas such as hospitals, schools, etc. To make the system carry out body temperature measurements to reduce the risk of infection, a remote measurement system combined with people identification is needed.

To achieve a remote method, non-contact infrared temperature measurement modules such as MLX90614 and MLX90615 are applied for this system. These modules can detect the surface temperature of an object; however, the accuracy of measurement is dependent on the cost and size of the infrared sensors. Additionally, the distance between the measurement and a person is largely influenced by the result. In consideration of the whole system, it first detects the face in the area of the attendance system before face recognition. There are mainly two ways to perform face detection: either using depth information to check whether a person’s face is in front of the camera, or using a single RGB camera with a face detection algorithm to perform face detection on the input image. Using depth information to activate face recognition, the overall speed is faster. However, as long as something passes through the camera to generate depth, the system will be activated. As a consequence, the accuracy will be poor, and the false detection rate will be higher. Using the face detection algorithm to detect faces in RGB images can improve the false detection rate, but the overall process speed is slow.

In this paper, a face recognition and temperature measurement system is proposed. It is designed as an attendance system for access control to instantly recognize faces by continuous image input and measure body temperature at the same time. The proposed masked face recognition and temperature measurement are constructed in the embedding system. It has the following key contributions:

- The proposed system specifically deals with the problem of low accuracy for people who wear masks. We simulate a mask on the VGGFACE2 [13] dataset and train FaceNet [14] to perform masked face recognition. The masked face recognition model achieves great accuracy on the LFW [15] and MFR2 [16] datasets.
- Face detection and recognition based on deep learning have been implemented on the embedded system of Raspberry Pi 4 to deal with real unconstrained scenes. By integrating a thermal imaging camera, we can perform face recognition and temperature measurement at the same time to assist in managing health status.
- We design a user interface (UI) to make the attendance system more convenient. There are three search methods to choose from, namely, search by date, search by month, and search by interval.

This paper is organized as follows. Section 2 discusses the related works. Section 3 discusses the proposed algorithm for face detection and recognition in the CNN approach. Section 4 describes the proposed attendance system with temperature measurement. Section 5 presents the system results and discussions. Section 6 concludes this paper.

## 2. Related Works

In this section, we will introduce the research on face detection and face recognition. To achieve a face recognition system, several steps are required, including image preprocessing, face detection, and facial feature vector extraction. Finally, the extracted vector is compared with the facial feature vectors in the database to perform face recognition.

### 2.1. Face Detection

Face detection is a key step before many face applications, such as face recognition, face verification, and face tracking. In the past few years, face detection methods have also developed quite well. The research on face detection is based on objection detection. The traditional object detection algorithm is commonly realized by combining a feature extractor, such as HOG, SIFT, or the Haar wavelet, and a classifier, such as SVM, linear regression, or decision tree [17,18]. A survey of face detection using hand-crafted features can be found in [19,20]. In recent years, convolutional neural networks have achieved great results in object detection and image classification, which have inspired face detection to achieve better results through convolutional neural networks. Li et al. [21] used a cascade structure and a convolutional neural network to improve the accuracy and speed of detection. At that time, it reached the highest score in the Face Detection Data Set and Benchmark (FDDB) [22].

A series of developments based on the region convolutional neural network (R-CNN) [23] was proposed, which uses a two-stage method to achieve the object detection task. R-CNN uses selective search to find 2000–3000 region proposals. Then, it resizes them to the same size and sends them into the CNN to retrieve features, and classifies them using SVM. After classification, linear regression is applied to the bounding box. Fast-RCNN [24] is proposed to improve R-CNN in that R-CNN calculates 2000 region proposals into the CNN, which requires the individual computation of many repetitive regions, while Fast-R-CNN only calculates the CNN once, and then uses the features extracted from the CNN for 2000 region proposals. Region of interest pooling (RoIpooling) is used to output the extracted regions to the feature map, and then each of them is connected to a fully connected (FC) network to perform softmax classification and bounding box regressor. R-CNN and Fast R-CNN use selective search, which is very time-consuming. Another improved version was proposed in [25], called Faster R-CNN, which directly uses a neural network to find a positive anchor to generate a region proposal. Compared to selective search, it is a great advancement. 

Although two-stage object detection algorithms have achieved great performance, they suffer some problems with long latency and low speed. Several researchers have improved object detection to single-shot models, such as YOLO [26], using CNN to predict multiple bounding boxes simultaneously and calculate the probability of an object for each box, and single-shot multi-box detector (SSD) [27], using convolutional layers of different depths to predict targets of different sizes, using lower layers with a larger resolution for small targets, which means that smaller anchors are set on the lower feature maps and larger anchors on the higher feature maps. An improved version of [23] was proposed, such as YOLOv3 [28], which introduced residual architecture and FPN [29] in the network architecture to improve the detection accuracy for small targets; YOLOv4 [30], which introduced CSPNet [31], SPP [32], and PAN [33] in the network architecture to improve computation speed while maintaining accuracy; and YOLO5Face [34], which improved the network architecture to improve accuracy in face detection. The widely used architecture, SSD, and other similar architectures predict bounding boxes on multiple layers [35,36,37]. These works execute the object detection with 30 frames per second (fps) or more on GPU. Compared to Fast-RCNN, the method proposed by [27] does not generate the region proposal but computes the features at once. As a consequence, it can improve the speed of object detection to achieve 59 fps with NVIDIA Titan X.

### 2.2. Face Recognition

There are two main directions of face recognition, which are holistic features and local features. Holistic features treat the whole face as a single feature for recognition. The local feature approach first identifies the local features on the face, usually the eyes, nose, and mouth. The individual results of the local features are combined to produce the final result, just like one can judge a person by the whole face, or sometimes by certain parts of the five senses.

Similar to face detection, face recognition has also been affected by the rapid development of CNNs in recent years. At present, most methods with high recognition rates use convolutional neural networks to extract features and then recognize them. First, DeepFace [38], developed by Facebook, set a benchmark far higher than the traditional method and opened the first feature extraction with the CNN. Its most special feature is the use of 3D models to align faces so that, afterward, the CNN can have the greatest effect. In DeepID3 [10], two very deep neural network architectures are proposed for face representation. These two architectures are constructed from the stacked convolution and inception layers proposed in VGGNet and GoogLeNet, making them suitable for face recognition. During training, joint face identification–verification supervisory signals are added to the intermediate and final feature extraction layers. The set of the two proposed architectures reaches an accuracy of 99.53% on the LFW dataset.

FaceNet was proposed by Google in 2015. Unlike previous face classification networks, which directly output classification results, FaceNet outputs quantified feature values. The detected facial features are then compared with the facial characteristics in the database to output the results. Because FaceNet only extracts features, the advantage of FaceNet over traditional face classification networks is that it does not need to retrain the network when new people are added to the database. The traditional face classification network requires fine-tuning the fully connected layer of the model for the model to recognize the new target. FaceNet is trained by a triplet loss, which is a type of ranking loss used to train samples with low variability. The triplet loss consists of an anchor, a positive sample, and a negative sample. It makes the distance between the anchor and the positive sample as small as possible and the distance between the anchor and the negative sample as large as possible. FaceNet achieves 99.63% on the LFW dataset.

Center loss was proposed by [39]. Center loss learns the center of the depth feature of each class while penalizing the distance between the depth feature and the corresponding class center. Softmax loss and center loss can increase inter-class dispersion and intra-class compactness at the same time. Both contrastive loss and triplet loss are used to improve the discriminative ability of deep features. However, when based on large-scale data sets, it needs to face the problem of huge sample pairs and sample triplets sampling. Center loss uses the same training data format as softmax loss and does not require complex training data resampling.

In real unconstrained scenes, there are still many challenges in the application of face recognition systems [40]. Even though ArcFace is powerful [41], it can only achieve an accuracy of 63.22% on the Real-World Masked Face Recognition Dataset (RMFRD) [42]. This result is based on [43] when ArcFace was not retrained on this dataset. Although CNN-based methods reach the goal of high accuracy in face recognition, they still fail to recognize people well when someone is wearing a mask. An intuitive approach is to use the dataset of masked faces to train the model. However, the problem with masked face recognition is the lack of a dataset, which can easily cause the model to overfit. Simulating masks on face datasets such as VGGFACE2 is a way to deal with this problem. In [16], a tool to mask faces effectively has been proposed. The tool can generate a large dataset of masked faces, and it can be used to train an effective face recognition system with target accuracy for masked faces.

## 3. Proposed Face Detection and Recognition Method

**Figure 1:**
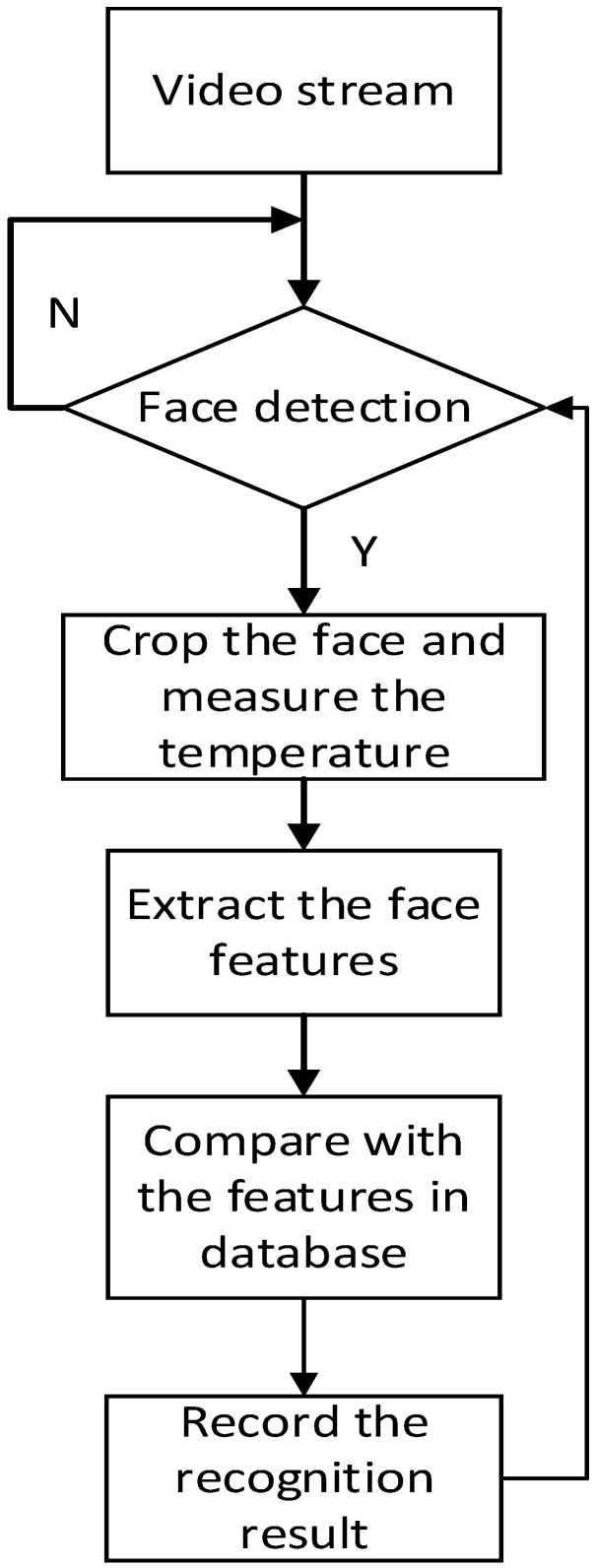
The execution flow of entire system.

Our goal is to realize an attendance system that can be managed through face recognition. To be a good attendance system, it must have several characteristics, namely a high recognition rate, fast recognition, and the ability to cope with many different faces. This work uses two-stage networks to reach the goal of face detection and recognition. The execution flow of the entire system is shown in Figure 1. First, the system will continuously detect the input screen. When a face is detected on the screen, the system will immediately crop the face and measure the temperature around the face area. Then, the cropped face image will be sent to the face recognition model to generate a facial feature vector, which will be compared with the facial features previously built into the database to confirm the identity of the person. Finally, the recognition results and the measured temperature are recorded. This method can be used to record the temperature of people while assisting them to punch in and out. In today’s rampant COVID-19 environment, it is important to measure and record the temperature of people entering and exiting.

### 3.1. Face Detection

To reach the goal of real-time face detection, we analyzed two kinds of methods as follows.

#### 3.1.1. Single-Shot Multi-Box Detector

The Single-Shot Multi-Box Detector (SSD) is a single-shot detector that uses a set of default boxes over different aspect ratios and scales to locate the bounding boxes. Additionally, the network combines predictions from multi-scale feature maps with different resolutions to naturally handle targets of various sizes. Low-level feature maps with larger resolutions can be used to detect smaller targets, and high-level feature maps can be used to detect larger objects. To make it run faster on an embedded system such as Raspberry Pi, the lite backbone network is adopted. It has only 8 convolution layers, and the total model has only 24 layers with the location and classification. The model can detect faces of general size, but for small or large pose changes, the detection effect is not as good as the big model. For an attendance system, people will always face the camera at a fixed distance and alleviate this problem in this case.

#### 3.1.2. Haar Cascade Classifiers

Although the lite version of the SSD can achieve real-time computing, the system still needs to connect with a complicated recognition network, which needs a lot of computation time. As a result, a lighter and faster detection network is required. Haar cascade classifiers were proposed by [44]. This is a machine learning-based method that trains the cascade equation through many positive and negative examples and then applies it to other images. It does not require complicated neural network calculations, so the calculation speed is very fast. As shown in Table 1, the processing time per frame of Haar cascade classifiers is around 0.075 s, which is faster than the SSD.

#### 3.1.3. Consideration of the Face Detection Algorithm

We adopt Haar cascade classifiers as our face detection method. Although the detection effect of the masked face is worse than the SSD, masked faces can also be successfully detected when the background is not complex. We further adopt a method of detecting the face in every three frames to reduce the burden on the system.

### 3.2. Face Recognition

For face recognition, the proposed system uses a feature extractor generated by a deep neural network. Only single-digit image data in the database can have a fairly accurate result. This part mainly refers to the algorithm proposed by [14]. The main concept of FaceNet is to define each identity in Euclidean space. The high-dimensional features calculated by the same identity need to be closer than the high-dimensional features of different identities to train a general-purpose model. The advantage of this architecture is that when the model is trained to build a local database, only one face image is used as the label of the database. Thus, there is no need to record different images from many angles. We set the threshold at 0.8 feature distance. If the feature distance between the input image and all the images in the database is greater than 0.8, the system will display “unknown”.

#### 3.2.1. Data Preparation

**Figure 2:**
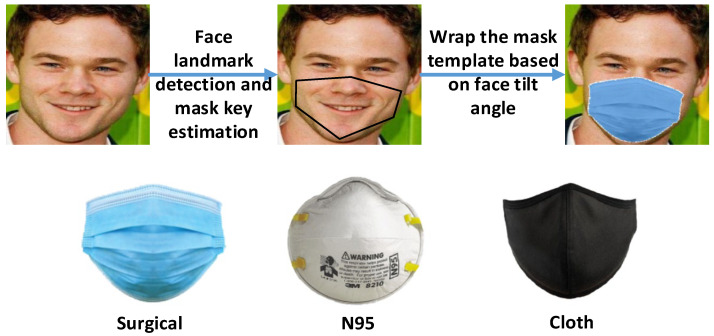
Masked face simulation flow and mask template. We used face landmark detection technology to synthesize different types of masks on the training images to increase the training data of masked face recognition model.

When a person wears a mask, the accuracy of FaceNet will decrease. One solution is to use a masked face dataset for training. However, the current dataset for masked faces is very scarce. We refer to [16] when mixing the simulated mask face into the training dataset and re-training. It provides source codes that can synthesize masks on human faces. It currently supports many different types of masks. We mainly used common surgical masks, N95, and cloth masks to synthesize the dataset. As a result, it has a better recognition effect under the condition of occlusion. The processing flow of simulating a mask is presented in Figure 2. It identifies all faces within an image and applies the user-selected masks to them by taking into account various limitations such as face angle, mask fit, and lighting conditions.

The proposed system uses this method to simulate a mask on the whole training dataset of VGGFACE2 and CASIA-WebFace [45]. VGGFace2 includes 3.3 M face images and 9131 identities, where each identity has more than 300 images. The dataset contains different times, makeup, accessories, expressions, etc., which are complex enough to train a network with great convergence. Based on this model, it can be derived from the real world. CASIA-WebFace is annotated with 10,575 unique people, with 494,414 images in total. When building a data set, it is easy to crawl the images from the Internet, but it is difficult to mark the images one by one. Therefore, the author used the IMDb website and completed the crawling and marking of images in a semi-automatic way.

#### 3.2.2. Training Setup

We used Inception-ResNet v1 as the backbone network and output a 512-dimensional feature vector. In the training stage, we used the simulated VGGFACE2 dataset to minimize the output feature gap of all the same people and maximize the gap between different people. The VGGFACE2 mixed with the simulated masked faces dataset was aligned and cropped using MTCNN [46] to obtain a tight bound on the faces in the images. The process avoids the redundant background in the image and puts more focus on the faces.

We preprocessed the image, which normalizes it to the Gaussian standard deviation. The preprocessing formula is shown as (1).

(1) $${Ｘ}_{white}=\frac{X-X_{mean}}{std\left(X\right)}$$

**Figure 3:**
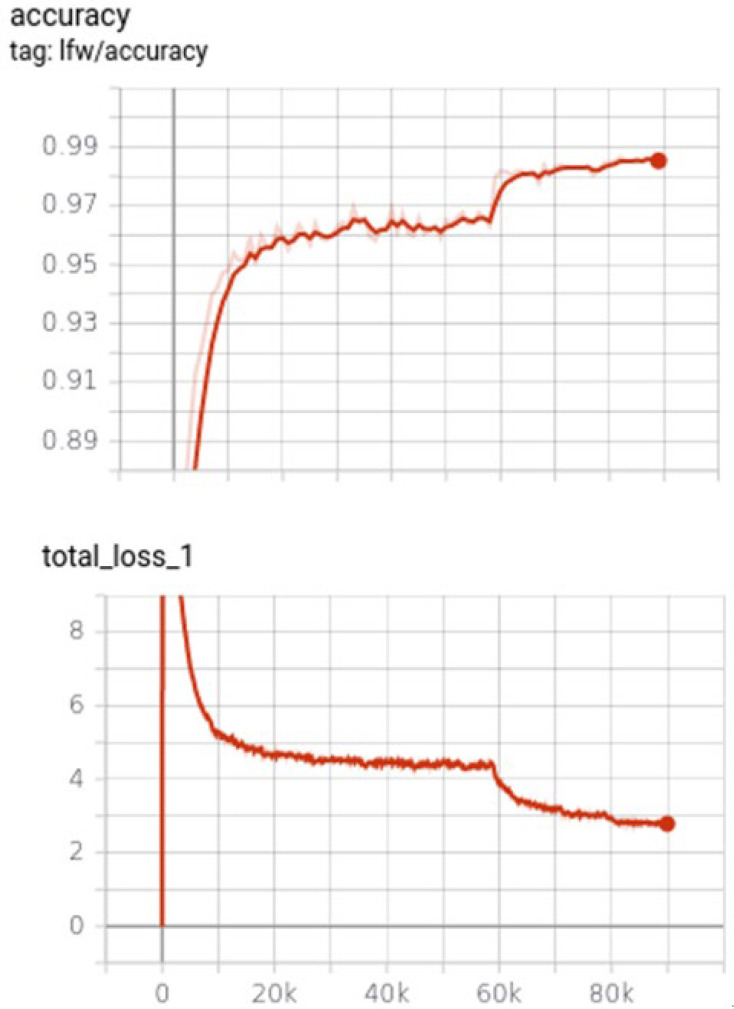
Our face recognition model was trained with 90 epochs in LFW dataset using different learning rates, showing the accuracy curves and loss curves in the training stage.

The purpose of this is to make the average of the input 0 and distribute it within a certain scale to better meet the needs of the convolutional layer of the neural network. In addition, for the training dataset, we used data augmentation to reduce the risk of overfitting. We trained 90 epochs using three steps of learning rates with values of 0.05, 0.005, and 0.0005 in different training stages. Finally, the model converges, and the accuracy of the LFW dataset is about 98%, as shown in Figure 3.

#### 3.2.3. Training Setup

To validate the training result, we used the LFW and MFR2 datasets. LFW includes 13,000 face images without the mask. MFR2 proposed in [16] provides a preprocessed dataset of real-world masked faces consisting of celebrities and politicians. Although the MFR2 dataset contains only 53 identities and only 269 images, it is an approach to show the effectiveness of the simulated masks dataset on real-world masked images. For the model trained on simulated VGGFACE2, an accuracy of 98% is achieved in both datasets, as shown in Table 2.

## 4. Overall Attendance System

The overall attendance system is designed with the face recognition system discussed in the last section. This section discusses the temperature measurement module and the integrated system design including the user interface.

### 4.1. Temperature Measurement

Infrared is an electromagnetic wave with a wavelength between microwave and visible light, and its wavelength is between 760 nm and 1 mm. From Stefan–Boltzmann’s law, we know that the total radiation emitted from the surface of an object is proportional to the fourth power of its temperature, so the temperature of an object can be calculated by measuring the total radiation energy. We analyzed two temperature sensors to decide on the proper one to combine with the proposed attendance system.

**Figure 4:**
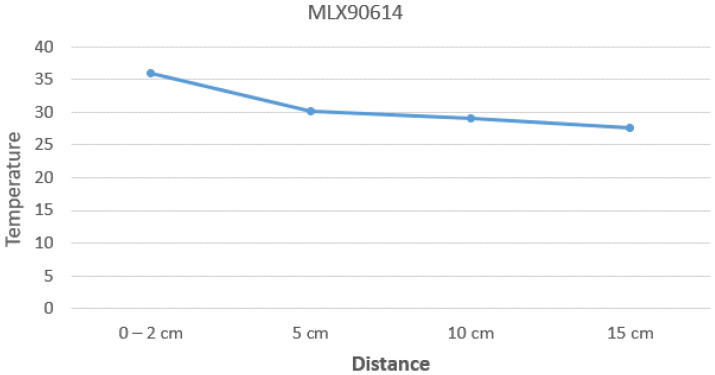
The effect of distance on temperature measurement.

We first investigated the non-contact temperature sensor chip, MLX90614 or MLX90615. It can detect the surface temperature of an object at a distance of about five centimeters with an error of about 0.2 degrees Celsius. It can be used in the appropriate position to detect the surface temperature of the object. However, MLX90614 measures the average temperature of the surface area of an object in the infrared circular projection area. If we want to obtain a more accurate measurement value, the measurement point area of the object under test cannot be larger than the surface area of the object under test; otherwise, the measurement value will be distorted. For example, when the distance is far, the projection on the surface of the object (such as the human face) may contain areas of lower temperature, such as glasses, hair, eyebrows, nose, and other parts. It will make the measured temperature lower than the real body temperature. Figure 4 shows the impact of the distance on temperature measurement. When the measurement distance is within 5 cm, the result is close to the real body temperature. However, when the measurement distance is out of 5 cm, the detected area may contain a low-temperature area, which makes the result unrealistic.

Then, we considered the other module, FLIR Radiometric Lepton 2.5. Most thermal imaging cameras can detect longer distances although the accuracy is lower, such as the thermal imaging module FLIR Radiometric Lepton provided by Raspberry Pi. The raw data received by Lepton 2.5 is 14 bits, and the value is in kelvin multiplied by 100.

The comparison of the two thermal sensors is shown in Table 3. Although the accuracy of the MLX90614 is higher than that of a thermal imaging camera, the temperature measurement distance must be very close. If MLX90614 is used as the temperature measuring device in our system, the person to be tested should be prompted to approach MLX90614 for temperature measurement very closely. It does not look friendly to the user. In our system, we use the FLIR Radiometric Lepton 2.5 for body temperature measurement.

In FLIR Radiometric Lepton 2.5, the temperature measurement error can be up to 5 degrees Celsius. Thus, we need to fine-tune the results according to environmental conditions such as room temperature and humidity. The measurement result can be expressed as (2).

(2) $$\mathrm{Temperature}\left({}^{\circ}C\right)=\frac{\mathrm{value}}{100}-273.15+\mathrm{offset}$$

The value detected by the thermal imaging camera will be first divided by 100, and then minused by 273.15 to transfer the value into degrees Celsius. Finally, the result will be fine-tuned with some offset according to the environment.

The proposed system converts the value from a thermal imaging camera into a Celsius temperature scale and then compensates the temperature according to the actual measured body temperature. The actual body temperature of different people is measured, and the required compensation value is calculated in the same environment. The final compensation result can make the temperature absolute value error about one degree Celsius.

### 4.2. User Interface

**Figure 5:**
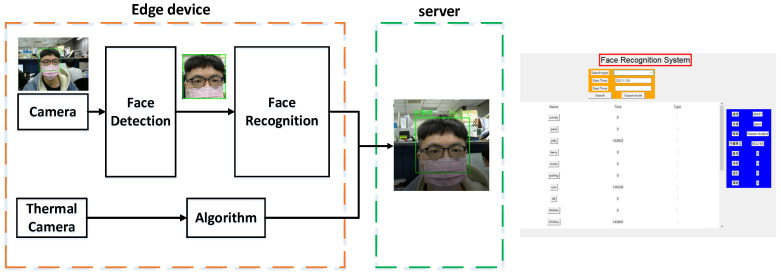
Architecture of masked face recognition system. With our user interface, it makes the entire attendance system more convenient.

For the application, we designed a personnel management system using a face recognition network. The attendance system is shown in Figure 5. The system consists of a UI and a MySQL-based database management system, which allows real-time monitoring of personnel data and matching of IDs in the data set. The qualified IDs will be sent to the database for storing information such as time and temperature, and the unqualified IDs will be put on the visitor list. In addition, the UI also includes a historical access mode, which can go to the database to retrieve the information of the relevant personnel to confirm their attendance status.

There are three kinds of query methods to choose from, namely, search by date, search by month, and search by interval. The search by date can output the time of the day when the person passed through the system, and the search by month/area will output the records of the month/area for each personnel’s leave, sick leave, etc. Additionally, clicking the orange export button in Excel can export Excel reports. Individual details can be displayed by clicking the name button in the data column in the blue box on the right.

**Figure 6:**
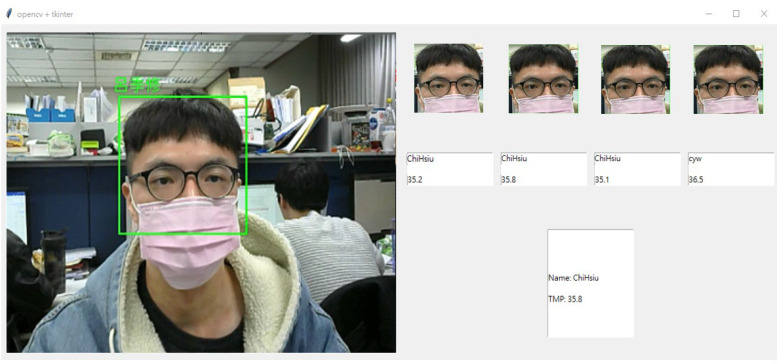
The diagram of our proposed face recognition attendance system. After matching with the database, the identity and temperature are recorded.

Figure 6 shows the face recognition execution system. The main screen is shown on the left, which contains the images captured by the webcam, face frames, and labels. The upper right and the middle right of the image will store the images and label data detected the previous few times, which can provide the information that the inspector loses due to the rapid movement of the inspected personnel. The bottom right of the figure is the currently detected tag data.

## 5. Results and Discussion

This part mainly discusses the implementation of the attendance system. Although the recognition model that we train on simulated VGGFACE2 has achieved a good accuracy and validation rate on both the LFW and MFR2 datasets, we still explore the chance of a lighter model backbone and different simulated training datasets. The real-world accuracy and the problem of the system will be discussed as follows.

### 5.1. Different Training Datasets and Lighter Model

We performed the same training process on CASIA-WebFace mixed with simulated masked faces to compare the performance. Then, we ran the validation on LFW and MFR2. Although the model trained on simulated CASIA-WebFace also achieves good accuracy results, the validation rate in MFR2 is quite low, as shown in Table 4. As a consequence, we chose VGGFace2 as the training dataset.

We also trained the small-scale models to see whether we can use the lighter model to achieve masked face recognition at the edge device. The results are shown in Table 5. We found that the lighter model such as SqueezeNet can maintain the accuracy on LFW, while the result on MFR2 is not as good as the deeper models. Although SqueezeNet and mobile SqueezeNet greatly reduce the number of parameters, as an attendance system, it requires high accuracy to ensure personnel control. Finally, we chose Inception-ResNet v1 trained on VGGFACE2 mixed with simulated masked faces to be the masked face recognition model in our system.

### 5.2. System Implementation and Results

**Figure 7:**
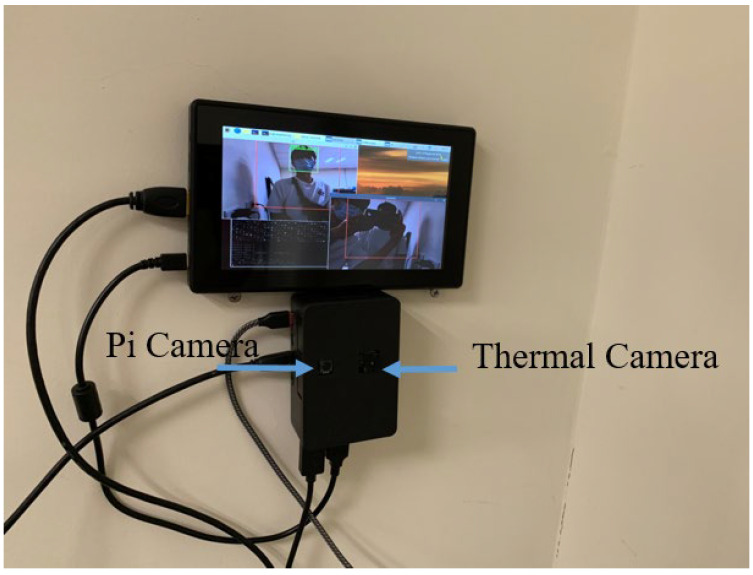
Embedded system settings, including Pi camera for video data input and thermal camera for temperature information input.

The architecture of the masked face recognition and temperature measurement system is shown in Figure 5. Before performing face recognition, we must first create a face database. We used face detection to frame the face position and took a photo to save it in the database. After all people had at least one image taken, the face was processed with FaceNet to perform the feature extraction, and the feature vectors were saved. The system is set up as a punch card system with temperature measurement. It detects and recognizes the human face through the edge device Raspberry Pi 4. We also performed the temperature measurement by the thermal imaging module FLIR Radiometric Lepton provided by the Raspberry Pi. Finally, the recognition result and body temperature data were sent back to the server. On the server side, the attendance time and the body temperature of each person were recorded. Through the integration of the above process, the system can achieve one stage of masked face recognition and temperature measurement. The edge part of the system was set up as shown in Figure 7. There was also a small screen on the edge device to present the recognition result and body temperature to the users.

### 5.3. Simulation Results

For the real-world experiment, we added 17 people to the database for testing purposes. Each person saved two images in the database: one with a mask and the other without a mask. Each person performed face recognition on different days, and the accuracy result after 20 times is shown in Table 6. The accuracy with a mask and without a mask is 94.1% and 91.8%, respectively. The result shows that the model trained by the simulated masked dataset can be successfully applied to a real-world situation.

We have some points to discuss on the problem. In actual application scenarios, some factors easily cause detection and recognition failure. Firstly, people wearing a mask with long bangs usually fail to be detected. Even if the face can be detected, the recognition accuracy is low. Secondly, if the color of the mask is the same as the background, the detection model will be hard to determine the correct position of the face for detection. Thirdly, those who wear glasses with color-changing lenses need to take off the glasses during file creation and recognition to prevent the color lenses from blocking the characteristic information of the face. Finally, if the face is too different from the database, it will reduce the face recognition result. For example, once a person does not wear glasses in creating the database, it may easily cause recognition errors when the person is wearing glasses.

## 6. Conclusions

Due to the recent impact of COVID-19, people are being encouraged to wear masks to prevent the spread of germs. This derives the proposed design using the non-contact methods for people attendance systems. Compared to fingerprints and irises, face recognition is accurate and does not require close contact, which significantly reduces the risk of infection. It is suitable for use as a punch card system during epidemic prevention carried out in major public places. The proposed system uses a non-contact method for attendance records to avoid unnecessary contact between people. To solve the problem of low accuracy on masked face recognition, we simulate the masks on the VGGFACE2 dataset and retrain FaceNet to perform masked face recognition. The masked face recognition model achieves great accuracy on the LFW and MFR2 datasets. Face detection and recognition have been implemented on the Raspberry Pi 4. Combining the system with a thermal camera, it can perform masked face recognition and temperature measurement at one time. The user interface is also made to make the overall system more convenient.

Our future research objective is to incorporate 3D face reconstruction into the system. By using only RGB images as input to generate 3D face models, better face recognition accuracy can be achieved.

## Figures and Tables

**Table 1:** Processing time of face detection on Raspberry Pi 4.

Face Detection Method	SSD	Haar Cascade Classifiers
Processing time	0.1605 s	0.0753 s

**Table 2:** Simulated VGGFACE2 verification on different datasets.

Dataset	Accuracy	Validation
LFW	0.9895	0.9616 @FAR = 0.001
MFR2	0.9834	0.8774 @FAR = 0.002

**Table 3:** Comparison of different temperature sensor.

Temperature Sensor	MLX90614	FLIR Radiometric Lepton
error	0.2 °C	5 °C
Measurement distance	5 cm	1 m
cost	low	high

**Table 4:** Simulated CASIA-WebFace verification on different datasets.

Dataset	Accuracy	Validation
LFW	0.9850	0.9180 @FAR = 0.001
MFR2	0.9669	0.6636 @FAR = 0.002

**Table 5:** Accuracy of different models trained on simulated VGGFACE2.

Model	Accuracy		Parameters
	LFW	MFR2	
Inception-ResNet v1	0.9895	0.9834	27.92 M
Inception-ResNet v2	0.9891	0.9846	59.55 M
SqueezeNet	0.9725	0.9457	6.19 M
Mobile SqueezeNet	0.9721	0.9492	5.90 M

**Table 6:** The accuracy of face recognition without and with a mask in the real world with simulated VGGFACE2.

Training Dataset	Accuracy without Mask	Accuracy with Mask
Simulated VGGFACE2	91.8%	94.1%

## Data Availability

Not applicable.
